# Physiological Hazard Assessment While Wielding Personal Protective Equipment (PPE) Among Health Care Workers

**DOI:** 10.7759/cureus.23510

**Published:** 2022-03-26

**Authors:** Jisa George, Ranjana Verma, Naseema Shafqat

**Affiliations:** 1 Medical Surgical Nursing (Oncology Nursing), Nursing College, All India Institute of Medical Sciences Bhopal, Bhopal, IND; 2 Cardiac/Thoracic/Vascular Surgery, Nursing College, All India Institute of Medical Sciences Bhopal, Bhopal, IND; 3 Obstetrics and Gynecology, Nursing College, All India Institute of Medical Sciences Bhopal, Bhopal, IND

**Keywords:** dermatological reactions, physiological hazards, ppe, personal protective equipment, health problems

## Abstract

Introduction

Accurate use of personal protective equipment (PPE) is an essential part of infection prevention and control measures to protect health care workers and patients from various hazardous conditions. Health care workers caring for patients with potentially infectious health conditions are using PPE for long periods of time. However, long-term use of PPE can cause many physiological health hazards among health care workers. So, the current study was carried out to assess the general health problems and dermatological problems experienced by health care workers with PPE use.

Materials and methods

A descriptive cross-sectional survey has been carried out in two selected tertiary care hospitals in central India by recruiting 301 health care workers. Non-probability convenient sampling technique was used to select participants for the study. Sociodemographic Performa and structured questionnaires were used to collect data on demographic characteristics of the participants and various health problems experienced by health care workers with PPE use. Collected data were analyzed using appropriate descriptive and inferential statistics.

Results

The current study reported excessive sweating (86.4%), difficulty in reading (85%), dry mouth (80.7%), and breathing difficulty (74.1%) as the most common problem associated with PPE use. In addition to this, adverse reactions like headache, restlessness, and dizziness were reported by 70.1%, 64.5%, and 50.8%. Indentation and pain on the back of the ears (76.1%), skin soaking (67.1%), and excessive sweating (76.1%) were identified as the most common problems related to N-95 masks, gloves, and coverall use.

Conclusion

The current study revealed a higher incidence of various health problems with PPE use among health care workers. The findings of the study highlight the importance of developing various guidelines to reduce the negative impact of PPE use and implementing preventive measures to decrease health problems associated with PPE use.

## Introduction

Personal protective equipment (PPE) is intended to ensure the workplace safety of health care workers and to protect them from occupational hazards and injury [1]. Proper use of PPE including N-95 masks/surgical masks/face shield/goggles, latex gloves, and coverall/gown is indispensable to protect against the physical, chemical, and biological factors encountered in the work environment. The use of PPE plays a vital role in infection control and preventive strategies to stop the transmission of potential pathogens and to protect health care workers and patients. Moreover, correct use of PPE is considered an important part of a comprehensive strategy for preventing the transmission of pathogens from patients to health care personnel (HCP). Adequate training and supervision are essential to ensure the correct use of PPE [2].

Infection prevention and control strategies and rational use of PPE have been suggested during the outbreak of viral pathogens that occurred in recent decades, such as severe acute respiratory syndrome coronavirus (SARS-CoV) in 2002-2003, Influenza Pandemic (H1N1) in 2009, and the Middle East respiratory syndrome coronavirus (MERS-CoV) in 2012 to protect health care workers from infections [3-5]. Therefore, consistent use of PPE was recommended during the COVID-19 pandemic for health care workers based on previous experience with other viral outbreaks. Besides, PPE is commonly used in a health care setting as standard or transmission-based precautions for the protection of the health care workers and to prevent the further spread of infection [6-9]. So as to prevent transmission of infection, health care workers are using PPE for long periods while engaged in patient care in the current scenario.

Prolonged periods of wearing PPE can cause various general health problems and dermatologic reactions due to friction, physical strain, sealing, and emotional issues such as fear of getting infections, isolation, and emotional burden [10,11]. PPE use has significantly affected the mental well-being of frontline health care workers. Stress, anxiety, and psychological claustrophobia are also reported by many users of PPE. Apart from this verbal communication difficulties, reduced tactile sensitivity and sense of smell, difficulties with fine hand movements, and impaired vision were also identified as topmost problems with PPE use [1,12]. However, very few studies have explored problems associated with PPE among health care workers in India. So, the current study was planned with an aim to assess various general health problems as well as dermatological problems with PPE use among health care workers.

## Materials and methods

A descriptive cross-sectional survey has been carried out from July to November 2021 among health care workers in two selected tertiary care institutions in central India selected through convenient sampling to assess the various health problems related to PPE use. Three hundred and one health care workers working in selected tertiary care institutes who met inclusion and exclusion criteria were selected by adopting a convenient sampling technique. Health care workers with more than six months of experience in the selected health care institutions, who can understand English or Hindi and are willing to participate were included in the study.

Data were collected using sociodemographic Performa to assess the demographic variables of the study subjects and a structured questionnaire with established validity and reliability to assess various problems faced by health care workers related to PPE use. All the data collection tools were developed by researchers based on available literature and the objectives of the study. Sociodemographic Performa included eight items to assess the demographic characteristics of the participants. Questionnaire to assess problems experienced by health care workers due to PPE use included 14 items related to general health problems and a total of 15 items to identify skin allergic reactions due to N-95 masks/surgical mask/ face shield/goggles (seven items), latex gloves (four items), and coverall/gown (four items). Content validity of the data collection tool was obtained from a panel of experts. Cronbach alpha was calculated to check for internal consistency and the value was found to be 0.87. The sample size for the study was estimated based on the findings of the pilot study using power analysis.

Ethical clearance for conducting the study was taken from the Institutional Human Ethics Committee of All India Institute of Medical Sciences (AIIMS), Bhopal, Madhya Pradesh, India (IHEC-LOP/2021/ IM0363). Written informed consent was taken from each participant prior to data collection. Permission to conduct the study was obtained from the concerned hospital authority. Data were collected from participants through electronic means using Google Forms. Invitation to participate in the study along with the link to the questionnaire was circulated to participants either through e-mail or What’s App mobile application. Collected data were analyzed using SPSS version 21 (IBM Corporation, Armonk, NY, USA) and the level of significance was set at 0.05 level. Appropriate descriptive and inferential statistics were used to analyze the data.

## Results

Final data analysis was carried out for 301 health care workers who participated in the study. The participants for the study included 81 (26.9%) doctors and 220 (73.1%) nursing personnel. Among doctors four (4.9%) were Professor/Associate professor/Assistant Professor, 76 (93.82%) were Senior Resident/Junior resident. However, among nurses, most of the participants 189 (85.9%) were nursing officers and 29 (13.18%) were senior nursing officers. The higher proportion of the participants 206 (68.4%) were males. While considering the area of work highest number of participants 143 (47.5%) were working in general wards followed by 72 (23.9%) in Intensive Care Units and only 29 (9.6%) were working in Operation theatres. The mean age of the participants was found to be 28.95±3.12. Most of the participants 222 (73.8%) were below the age of 30 years and only three participants were above the age of 40 years. The highest number of participants 160 (53.2%) had an experience between one and five years and only 13 (4.3%) had an experience of more than 10 years with a Mean ± SD (standard deviation) of 3.90±3.10.

The majority of the participants, 271 (90%) received training related to PPE use. One hundred and seventy (56.5%) of the participants revealed that they experienced problems related to PPE use. Among health care workers who have experienced problems due to PPE use, 128 (42.5%) had health problems only after the continuous use of PPE for more than four hours. Table 1 explains general health problems related to the use of different types of PPE by health care workers. The most common general health problems experienced by the health care workers were excessive sweating (86.4%), difficulty in reading (85%), dry mouth (80.7%) breathing difficulty (74.1%), and difficulty in walking (71.4%).

**Table 1:** Various health problems experienced by health care workers related to PPE use.

S. No	Health problems	Yes	No
1	Breathing difficulty	223(74.1%)	78(25.9%)
2	Nausea	107(35.5%)	194(64.5%)
3	Weakness	159(52.8%)	142(47.2%)
4	Restlessness	194(64.5%)	107(37.5%)
5	Leg cramps	121(40.2%)	180(59.8%)
6	Sleep deprivation	108(35.9%)	193(64.1%)
7	Cervical pain	121(40.2%)	180(59.8%)
8	Dry mouth	243(80.7%)	58(19.3%)
9	Excessive sweating	260(86.4%)	41(13.6%)
10	Dizziness	153(50.8%)	148(49.2%)
11	Headache	211(70.1%)	90(29.9%)
12	Difficulty in walking	215(71.4%)	86(28.6%)
13	Difficulty in reading	256 (85%)	45 (15%)
14	Impaired cognition	110(36.5%)	191(63.5%)

**Figure 1:**
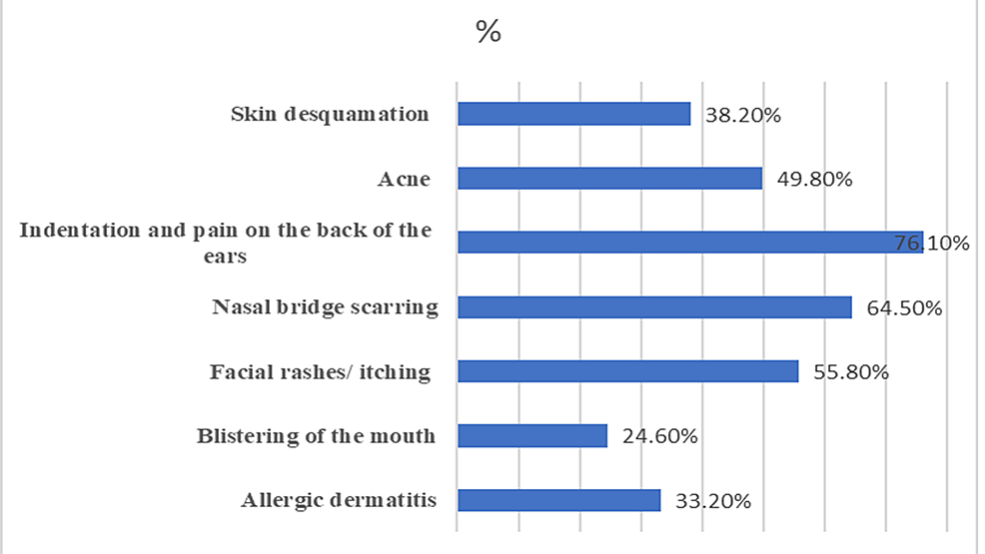
Dermatological problems with surgical mask/ face shield/goggles use

A large number of participants reported indentation or pain on the back of the ears (76.1%) and nasal bridge scarring (64.5%) related to the N-95 mask. Skin soaking (67.1%) and excessive sweating (76.1%) were the major problems among health care workers due to latex gloves and coverall use, respectively. Figure 1 and Table 2 describe allergic skin reactions related to various types of PPE among health care workers. In the present study, no significant association was found between health problems related to PPE use among health care workers with sociodemographic and other baseline variables. 

**Table 2:** Dermatological problems related to latex gloves and coverall/gown use among health care workers.

S. No	Health problems	Yes	No
		f (%)	f (%)
	Related to latex gloves		
1	Skin itching/rashes	157 (52.2%)	144(47.8%)
2	Contact dermatitis	94 (31.2%)	207 (68.8%)
3	Dry skin	180 (59.8%)	121 (40.2%)
4	Skin soaking	202 (67.1%)	99 (32.9 %)
	Related to coverall/gown		
1	Skin itching/rashes	119 (39.5%)	182 (60.5%)
2	Contact dermatitis	66 (21.9%)	235 (78.1%)
3	Dry skin	107(35.5%)	194 (64.5%)
4	Excessive sweating	229 (76.1%)	72 (23.9%)

## Discussion

Infection prevention and control strategies are very critical in preventing potential exposure to pathogens and protecting health care workers from infections. In addition to infection prevention and control measures, judicious and rational use of PPE play a fundamental role in ensuring the safety and protection of health care workers. But, prolonged periods of wearing PPE were significantly associated with many health problems among health care workers. The current study carried out among health care workers identified various health problems related to PPE use.

In our study, the most common problem associated with PPE wielding was excessive sweating (86.4%), difficulty in reading (85%), dry mouth (80.7%), and breathing difficulty (74.1%). Additionally, presenting features of dehydration, such as restlessness (64.5%), dizziness (50.8%), and muscle cramps (40.2%), were reported by health care workers. Consistent findings have been reported in studies conducted in India [1,10]. Problems with heat stress and strain are profound and more challenging for the users of PPE which can exacerbate physiological strain and can lead to decreased physical and cognitive performance. In tropical countries like India during the summer season, the problems with PPE use were worse for health care workers especially when facilities for controlling environmental temperatures such as the central air conditioning system was shut for preventing the spread of infection. Drinking large quantities of cold water before donning, drinking ice slurry, and frequent change of PPE were adopted by health care workers to decrease dehydration. Likewise, providing constant ventilation was found to be an effective strategy to decrease heat stress [1,10,13].

In addition to this, prolonged periods of wearing PPE can lead to giddiness, discomfort, and headache due to heat and moisture produced inside the PPE. In the current study, 70.1%, 64.5%, and 50.8% reported headache, restlessness, and dizziness respectively which was concurrent with findings of previous studies [1,14]. Long periods of PPE use were associated with sleep deprivation and other sleep problems. Incorrect and inappropriate size of the PPE restricted movement of health care workers and they experienced difficulty in performing various procedures. Similar findings of difficulty to perform autopsies with PPE were found in a study carried out in India [1]. Another study carried out in India among frontline nurses during the COVID-19 pandemic also identified similar problems with PPE [15]. Most of the health care workers developed de novo PPE-related headaches and exacerbation of existing headaches in a study carried out in Singapore [16].

PPE such as N-95 mask, gown, gloves, and coverall worn for long periods of more than four hours may cause severe allergic skin reactions. N-95 mask or surgical mask is used to prevent the spread of respiratory infections. It is considered an effective strategy to prevent the spread of respiratory pathogens if used correctly. Indentation and pain on the back of the ears (76.1%), nasal bridge scarring (64.5%), and facial rashes and itching (55.8%) were reported as the most common dermatologic reactions related to N-95 mask, goggles, or face cover by the health care workers. Similar findings were conveyed in previous studies done in China and India [11,14,15,17]. Long periods of wearing N-95 masks lead to device-related pressure injuries on the skin behind the ear [18]. A study carried out in Singapore during the SARS outbreak identified facial itching, acne, and pigmentation around the nasal bridge and chin as the most common adverse reactions with PPE [19]. Comparable findings of increased incidence of acne were noticed among health care workers in the current study. Another study reported massive degree of facial skin irritation and other physical problems like dizziness and headaches with Filtering face-piece respirators (FFRs) [20,21]. Hot and humid climate created in the areas covered by masks and occlusion of pilosebaceous duct due to local pressure predisposes to flaring of acne [19]. Worsening episodes of asthma were also noticed with the use of the N-95 mask in the present study. A significant impact of the N 95 respirator mask on cognitive impairment leading to severe errors while performing tasks was evident from previous studies [22]. Moreover, the use of N-95 mask was associated with health problems like headache, nausea, vomiting, and giddiness due to hypoxemia, hypercapnia, and mechanical factors or the stress associated with its use [19,23]. Routine cleaning and moisturization of skin with emollients at least one hour before using facial PPE, wearing of facial PPE correctly, and selection of correct size are recommended as some strategies to prevent adverse events related to facial PPE [24].

Health care personnel can be contaminated with direct contact with patient skin or body fluids and indirect contact with the patient environment. Contaminated hands of the health care workers can be a source of infection and full protection with appropriate use of gloves were recommended during the COVID-19 pandemic. The most common adverse reactions experienced by the health care workers with the use of gloves were skin soaking (67.1%), dry skin (59.8%), and skin itching (52.2%). Consistent findings have been reported in studies carried out in India among frontline nurses [15] and among health care workers in China [11]. Concurrent findings have been reported in studies conducted during SARS in the year 2006 [19]. If the gloves are worn for long periods of time, the sweat evaporation is decreased and skin is prone to impregnation, contact dermatitis, and rashes as the hands are left in an air-impermeable environment. Besides this frequent hand hygiene also damages the skin barrier and causes dryness, itching, irritant contact dermatitis, and eczema. Measures like adequate cleaning and moisturization to maintain the skin barrier, wearing plastic gloves inside the latex gloves, and appropriate drying of hands before putting gloves can be adopted to reduce the severity of skin reactions and damage due to gloves [11,18,24,25].

Health care workers were using protective clothing to safeguard themselves from aerosols and other harmful substances. So, adverse reactions due to protective clothing have developed among health care workers who were using this for long hours. Health care workers reported excessive sweating (76.1%), skin rashes and itching (39.5%), and dry skin (35.5%) as major problems associated with the use of coverall or protective clothing. Consistent findings have been identified in previous studies to assess adverse reactions due to PPE during the COVID-19 pandemic [11,15]. Long periods of wearing coverall or protective clothing and it is getting humid due to excessive sweating have been documented as possible reasons for adverse reactions. Frequent change of protective clothing has been identified as the best alternative to reduce this problem [11]. It is very important to use appropriate levels of PPE as per the recommendations of regulatory bodies and government agencies to ensure adequate level of protection and to prevent the further spread of infection [26]. Moreover, device-related pressure injuries and skin tears have been reported among many health care workers due to wearing PPE. A survey carried out among health care workers found a high prevalence (42.8%) of device-related injury with PPE use among health care workers [27,28]. Device-related injuries were strongly correlated with the wearing time of the device. Therefore, it is important to make recommendations for frequent replacement of staff wearing PPE for long periods.

The current study investigated the negative impact of long-term wielding of PPE among health care workers. The findings of the study throw light on the importance of having guidelines for reducing the negative effects of prolonged use of PPE. Policies and protocols must be developed to minimize the continuous wearing of PPE for long periods of time. Especially, policymakers and administrators must implement appropriate control measures with positive reinforcement and feedback to reduce the negative effects of long-term use of PPE by ensuring proper standard and quality of PPE. Adequate training and supervision have to be provided to manage various problems with PPE use. However, the present study has limitations as the researchers assessed adverse effects related to PPE use merely on the basis of self-reported measures rather than observation and verification of the symptoms. Likewise, investigators could not assess the severity, location, and the number of incidences of adverse reactions due to PPE and also the role of multiple exposure factors on adverse health hazards related to wielding of PPE among health care workers. Large multicentric studies can be carried out in the future using more objective assessment strategies to identify and alleviate the adverse reactions to PPE use.

## Conclusions

The current study carried out among health care workers found a high incidence of many physical health problems and dermatological changes and reactions due to PPE use. The findings of the study highlight the importance of adopting various preventive measures to decrease health problems associated with PPE use. It is very important to take measures to mitigate the adverse effects related to the long-term use of PPE to face further waves of COVID-19 infections and new pandemic outbreaks in the future.
